# Design and modeling of a planar graphene structure as a terahertz cyclotron radiation source

**DOI:** 10.1038/s41598-021-95502-9

**Published:** 2021-08-05

**Authors:** Jordan Planillo, Fabio Alves

**Affiliations:** Naval Postgraduate School, 1 University Circle, Monterey, CA 93943 USA

**Keywords:** Physics, Nanoscale devices, Nanoscience and technology, Graphene, Electronic properties and devices, Electrical and electronic engineering, Lasers, LEDs and light sources, Other photonics

## Abstract

With incredibly high carrier mobility and saturation velocity, graphene would be an ideal candidate for a miniaturized solid-state cyclotron radiation source. A planar semicircular graphene arc geometry was investigated for emission in the 0.5–1.5 THz range. Analytical studies, confirmed by finite element simulations, show that the emitted THz frequencies are inversely proportional to the arc radius given a fixed charge-carrier velocity. The simulations show that the desired frequency spectrum can be obtained with design radii ranging from 50 to 150 nm. Interestingly, the radiated spectrum is independent of the frequency of the stimulation of the graphene nano-arcs. The simulations also indicate that the total output power correlates well with the Larmor formulation. The device is expected to emit 1 nW/cm^2^, which confirms the findings of existing research in this field. Such a design could yield a scalable and cost-effective THz source.

## Introduction

Sixteen years since its discovery^1–3^, graphene has been touted as a super material known for its excellent mechanical, thermal, and electrical properties. Promising applications range from a new construction material^4^ to a room temperature superconductor^5^. While most of the electronics applications for graphene involve its use as a field effect transistor to eventually replace silicon for computing applications, few have explored its use in radio frequency (RF) emissions^6^. Much of graphene’s RF applications have involved design and construction of conventional RF components such as transmission lines, waveguides^7^, and antennas^8,9^. More recently, graphene’s high carrier mobility^10^ and saturation velocity^11^ has been explored for its application as a solid state implementation of a free electron laser (FEL) and associated devices such as the wiggler^12,13^.

The use of graphene in RF emitter applications is primarily due to its high carrier mobility, high saturation velocity, and its tunable band gap. Highly conventional approaches and applications recreate existing RF structures in grapheme—namely the patch antenna^8^. The monolayer thickness of graphene makes it highly desirable for compact form factor applications. In another such application, a graphene substrate is used to enhance the response and tunability of plasmonic antennas^9^. By applying a back-gate field to the graphene substrate and altering the graphene’s bandgap the antenna can respond to a much wider span of incident wavelengths compared to the same photoconductive structure without graphene.

Less conventional RF emitter applications envision graphene as a substrate which can mimic the behavior of vacuum-based particle accelerator devices such as the cyclotron, FEL, or wiggler. Models of corrugated graphene show that under a constant DC bias, electromagnetic radiation can be emitted with a frequency equal to the carrier drift velocity divided by the corrugation period^12^. With this approach, emissions at THz frequencies are possible that are not dependent on the existence of THz switching technologies. Experimental implementations of corrugated graphene have shown that THz emission is possible^13^. Such an approach involves etching the periodic trenches onto a Ge substrate and transferring the graphene onto the patterned substrate. The graphene contours the substrate resulting in the desired corrugation. Expected power output for this device ranges from 1 pW/cm^2^ to 10 nW/cm^2^.

Other cyclotron based radiation methods involve radiative transitions between Landau levels in graphene^14–16^. Such methods subject graphene to a magnetic field such that quantized cyclotron orbits via the quantum Hall effect occur. Optical stimulus, by means of a laser, pumps electrons from the valence band to the conduction band of the graphene at energies set by graphene’s dispersion relation and by the Landau levels. Photon emission via transition between Landau levels occurs at far-infrared (FIR) and terahertz frequencies.

In this paper, the application of graphene as a miniaturized solid-state cyclotron radiation source in the form of semicircular arcs is explored. A design geometry should satisfy the desired frequency output range from 0.5 to 1.5 THz while being scalable with existing semiconductor processing methods. Modeling begins with a simple rotating dipole model as an idealized scenario for the device to obtain estimates for the power spectrum and radiation pattern. A finite element model that better represents the actual structure and allows for finite width and electronic contacts is also developed. To facilitate future device scalability, design requirements for higher density device arrays are considered.

**Figure 1:**
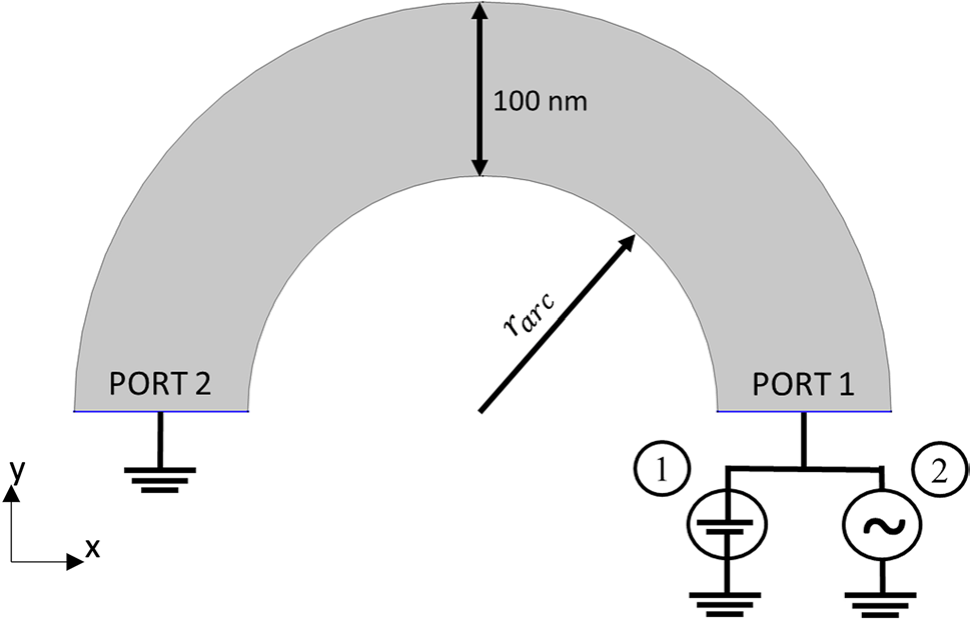
Schematic setup of the graphene arcs. In the dipole model, all of the arc’s charges are concentrated at a single point and traverse a semicircular trajectory at a radius r_arc_. The motion of the charges is expected to produce cyclotron radiation at frequencies inversely proportional to r_arc_. For the finite element DC simulation (1), a constant 1 V was applied. For the finite element RF simulation (2), signals at 4 GHz, 10 GHz, and 40 GHz of 1 V amplitude were applied to port 1.

We propose here a device that lies on a flat substrate plane in which graphene is patterned as a semicircular arc. Such a device will produce cyclotron radiation by charges traversing along the arc. Such a structure would be highly compatible with existing semiconductor processing techniques, especially in terms of scalability and high-density layouts (Fig. 1). Structures like this are now feasible since graphene manufacturing processes are mature enough to where whole wafers of single layer graphene (grain size ~ 10 um) can be purchased^17^. To model such a device and obtain performance estimates, we begin with a first principles equivalent analytical model in which the graphene device is approximated as a rotating electrical dipole^18^. In the dipole model, all the available charge carriers in the semicircular arc are concentrated in a singular point charge at the arc’s inner radius (${r}_{arc}$). Next, it is assumed that the singular point charge moves at graphene’s saturation velocity (${v}_{sat}$) of $4.25\times {10}^{7}$ cm/s^11^. For a sufficiently large and spontaneously applied field, the charge carriers in the graphene will form a shockwave spanning the width of the graphene sample^19^, hence a sufficiently short width would give the appearance of a moving point charge.

While graphene’s saturation velocity offers superior speed compared to most semiconducting materials, this velocity is still nowhere close to the relativistic speeds in free electron laser, wigglers, or synchrotron light sources^20^. This implementation of a solid state cyclotron radiation source operates in the non-relativistic regime. When reduced to this classic problem, this device is expected to emit radiation at a frequency equivalent to its angular velocity at a power given by the Larmor formula

1 $$P = \frac{{q^{2} a^{2} }}{{6\pi \epsilon_{0} c^{3} }}$$

where *q* is the effective single point charge of the graphene arc, *a* is the charge’s centripetal acceleration ($a = v_{sat}^{2} /r_{arc}$), $\epsilon_{0}$ is the vacuum permittivity, and $c$ is the speed of light in vacuum.

**Figure 2:**
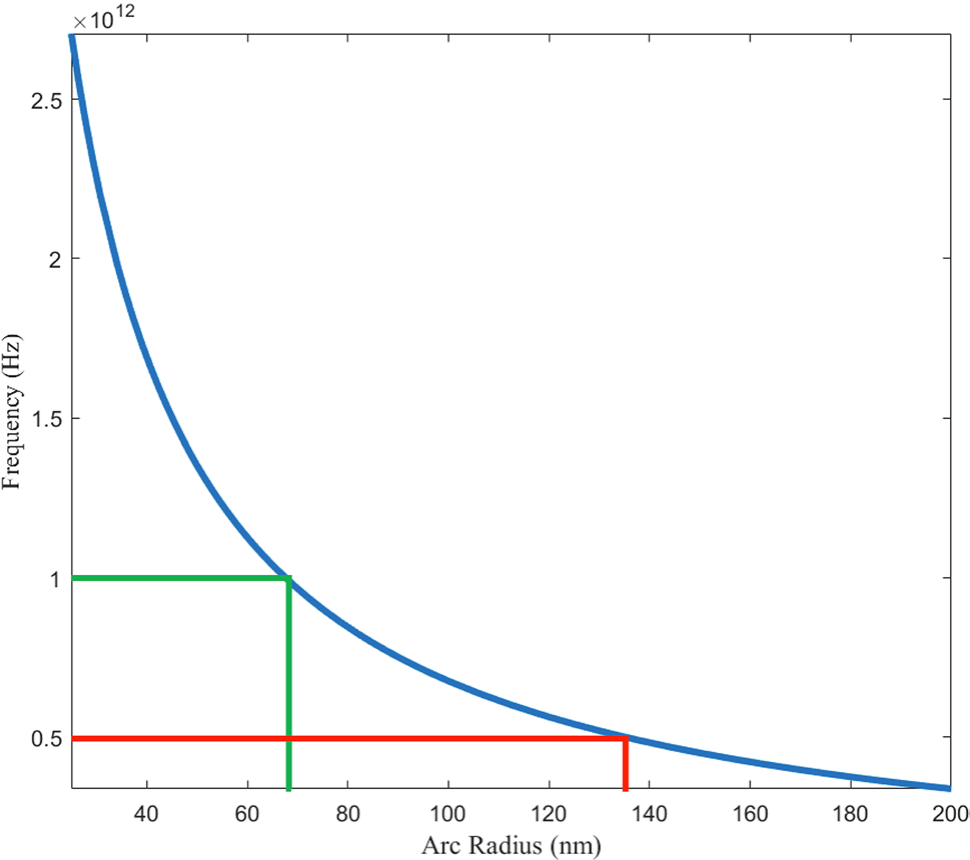
Trendline of emitted frequency vs. arc radius for a rotating dipole with a tangential velocity of ${v}_{sat}$ = 4.25 × 10^7^ $\text{cm/s}$. The target frequencies of 0.5 THz and 1.0 THz require an arc radius of 135 nm and 67 nm respectively.

For fixed particle velocity v_sat_ and frequency f, the required arc radius is r_arc_ = v_sat_/2πf. The relation of r_arc_ to frequency is displayed in Fig. 2. For design frequencies 0.5 THz and 1.0 THz they are 135 nm and 67 nm respectively. An arc width of 100 nm is chosen as it is sufficiently wide to avoid quantized current behaviors in the graphene nanoribbon regime^21,22^. These parameters can be easily manufactured with existing semiconductor processing methods while also being within the constraints of the current state of the art of graphene manufacturing. Such a device must fit within a grain of graphene, nominally 10 $\mu$ m× 10 $\mu$m. To ensure that the assumption of uniform circular motion is valid, arc lengths must be less than graphene’s mean free path, nominally ~ 1–2 $\mu$m at 293 K at carrier concentrations of ${10}^{12}$ cm^−2^^10,23^.

For a fabricated device, edge roughness will be present regardless of the process used to pattern the graphene and will be an additional source of scattering^21^. Such scattering will result in slower transit times through the arc and a much more broadly spread charge distribution in the arc. Consequently, the device will have reduced performance in terms of reduced power output, lower radiated frequency, and a radiation pattern which deviates from the ideal case. To mitigate the edge effects while maintaining the desired orbital radius, a fabricated device will need an additional margin on the interior radius to keep the desired orbital radius clear of edge defects and subsequent scattering. The size of this margin at minimum would be proportional to the average edge roughness for a given manufacturing process and at maximum the beam waist for the given process.

For a rotating dipole, the solutions to Maxwell’s equations yields the following radiated fields in spherical coordinates:

2 $$\begin{aligned} \vec{E} & = \frac{{\mu_{0} p_{0} \omega }}{4\pi r}\left\{ {\cos \left( \theta \right)\left[ {\cos \left( {\omega \left( {t - \frac{r}{ c }} \right)} \right)\cos \left( \phi \right) + \sin \left( {\omega \left( {t - \frac{r}{ c }} \right)} \right)\sin \left( \phi \right)} \right]\hat{\theta }} \right. \\ & \quad \left. { - \left[ {\cos \left( {\omega \left( {t - \frac{r}{ c }} \right)} \right)\sin \left( \phi \right) + \sin \left( {\omega \left( {t - \frac{r}{ c }} \right)} \right)\cos \left( \phi \right)} \right]\hat{\phi }} \right\} \\ \end{aligned}$$

3 $$\begin{aligned} \vec{B} & = \frac{{\mu_{0} p_{0} \omega }}{4\pi rc}\left\{ {\left[ {\cos \left( {\omega \left( {t - \frac{r}{ c }} \right)} \right)\sin \left( \phi \right) + \sin \left( {\omega \left( {t - \frac{r}{ c }} \right)} \right)\cos \left( \phi \right)} \right]\hat{\theta }} \right. \\ & \quad \left. { + \cos \left( \theta \right)\left[ {\cos \left( {\omega \left( {t - \frac{r}{ c }} \right)} \right)\cos \left( \phi \right) + \sin \left( {\omega \left( {t - \frac{r}{ c }} \right)} \right)\sin \left( \phi \right)} \right]\hat{\phi }} \right\} \\ \end{aligned}$$

where $p_{0}$ is the dipole moment, ($p_{0} = q \cdot r_{arc}$), ω is the charge’s angular velocity ($\omega = v_{sat} /r_{arc}$), $\mu_{0}$ is the vacuum permeability, $t$ is the time parameter; $r$, $\theta$, and $\phi$ are the respective radial, altitude, and azimuth coordinates with corresponding unit vectors $\hat{r}$, $\hat{\theta }$, and $\hat{\phi }$.

To assure that charges move along the proposed semicircular trajectory, the device is subject to high frequency and high field biases and will be operating in the non-equilibrium transport regime^24^. Such operating conditions can be reasonably achieved for conducting materials that do not have an abundance of charge carriers such as semiconductors or semimetals, like graphene^25^. Under these conditions, there is a noticeable imbalance in the number of charges entering and exiting the source and drain, hence leaving the device with a net charge. Additionally, the injected charges will form a shockwave which propagates along the arc at the saturation velocity^19^. The shockwave, or more importantly the shockwave front, which by nature is a highly concentrated collection of charge in conjunction with the constraints from arc’s width will give the appearance of a point charge. The arc length of the graphene arc should be less than the mean free path as to reduce the effects of scattering mechanisms. Longer lengths will adversely affect the uniform circular motion required in rotating dipole model. In addition, the rotating dipole model has to be modified to accommodate the semicircular trajectory of the carriers—unlike the circular motion of the classical problem.

For simple cyclotron radiation, the circular geometry is the most common. To accommodate the source and drain terminals for the solid-state implementation, a semicircular arc is chosen as this guarantees that the source and drain are maximally separated by a distance equivalent to the circle’s diameter. Such a design choice is preferable from a fabrication standpoint as both longer and shorter arc lengths would have sharp features that may not be reproduced correctly when fabricated. Additionally, the maximal separation reduces parasitic capacitances between the source and drain terminals. Having constrained the design to a semicircular arc, the transit time of the charge carriers is valid only for one half of an orbital period in the classic rotating dipole problem. To understand the behavior of this now modified problem, Eqs. (2) and (3) are multiplied by a window function $$\left[ {H\left( t \right) - H\left( {t - {\raise0.7ex\hbox{$T$} \!\mathord{\left/ {\vphantom {T 2}}\right.\kern-\nulldelimiterspace} \!\lower0.7ex\hbox{$2$}}} \right)} \right]$$, where $$H\left( t \right)$$ is the Heaviside step function. A Fourier transform of the modified classical solution is calculated over the valid times of 0 to T/2 where T is the orbital period in the classic problem. It is anticipated that this modified problem will not radiate at a single frequency like the classic problem in which a Fourier transform yields a Dirac function at the angular velocity. Instead, a spreading of the frequencies is expected about the angular velocity of the rotating dipole. If that is the case, then the shape of the spectrum would need to be determined in addition to the frequency that yields the most power. A dimensionless and reparametrized expression for the Fourier transformed fields is given below (4) where $$x$$ is the ratio of the frequency parameter $$\omega$$ to the charge’s angular velocity $$\omega_{0}$$. Given the equivalence of the electric and magnetic fields by multiplication of an orthogonal unit vector and a factor of the speed of light, depending on the unit system – only the electric field is shown.

4 $$\vec{E}\left( {x,\phi ,\theta } \right) = \frac{{\left( {1 + e^{ - i\pi x} } \right)\left( {\cos \phi - ix\sin \phi } \right)\hat{\phi } + \cos \theta \left( {1 + e^{ - i\pi x} } \right)\left( {\cos \phi + ix\sin \phi } \right)\hat{\theta }}}{{x^{2} - 1}}$$

An expression for dimensionless Fourier transformed Poynting vector $\vec{S} = \vec{E} \times \vec{H}$ can be written as follows:

5 $$\vec{S} = \frac{{2\left( {1 + \cos \left( {\pi x} \right)} \right)\left[ {x^{2} \left( {1 - \cos^{2} \phi \sin^{2} \theta } \right) + 1 - \sin^{2} \phi \sin^{2} \theta } \right]}}{{\left( {x^{2} - 1} \right)^{2} }} \hat{r}$$

**Figure 3:**
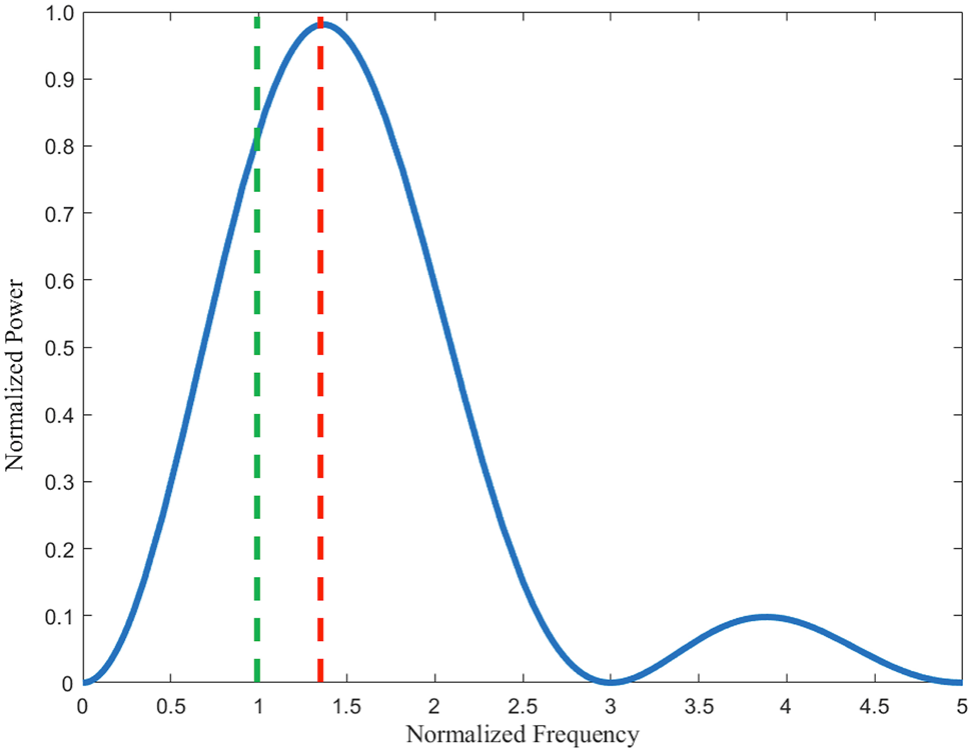
Calculated frequency spectrum for the modified rotating dipole lasting ½ of a full orbital period. The peak emission occurs at a normalized frequency at ~ 1.36. Another peak occurs at ~ 3.6.

**Figure 4:**
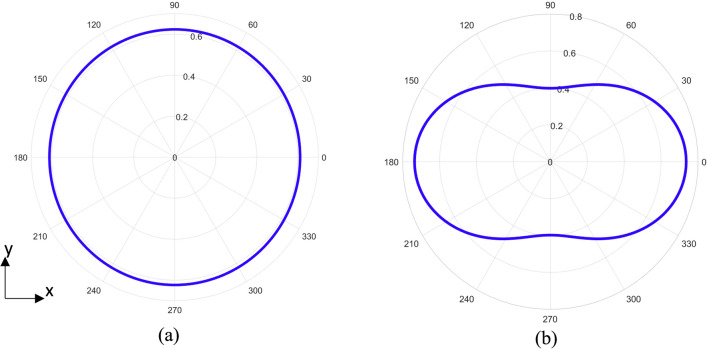
Calculated radiation patterns for the modified rotating dipole lasting ½ of a full orbital period (arbitrary scale) in the orbital plane. (**a**) Radiation pattern for normalized frequency equal to 1. The circular pattern is identical to the steady state rotating dipole in which the emitted frequency and angular velocity are equivalent. Power is distributed uniformly in the plane of rotation at this frequency. (**b**) Radiation pattern for normalized frequency equal to ~ 1.36. Power is distributed in all directions of the orbital plane, but is more biased along the x-axis.

From the dimensionless Poynting vector, critical values for this expression are sought to understand how the power output S is related to normalized frequency $\omega /\omega_{0}$. A numerical evaluation of the Poynting vector expression yields a maximum at $x$ ≈ 1.36 (Fig. 3). The resulting pattern radiates in all directions but is more biased along the x-axis (Fig. 4). For $x$  = 1, the expression for the dimensionless Poynting vector yields the same value everywhere in the horizontal plane—a result identical to the unmodified problem. Lastly, for frequencies ranging from $0 < x < 1$ the classic dipole shape is recovered in which the lobes are oriented along the x-axis and no radiation along the y-axis. Another maximum is observed at $x$ ≈ 3.6, however there will not be a large contribution at this frequency or any other frequencies beyond the first peak at $x$ ≈ 1.36.

## Results

### Dipole model

**Figure 5:**
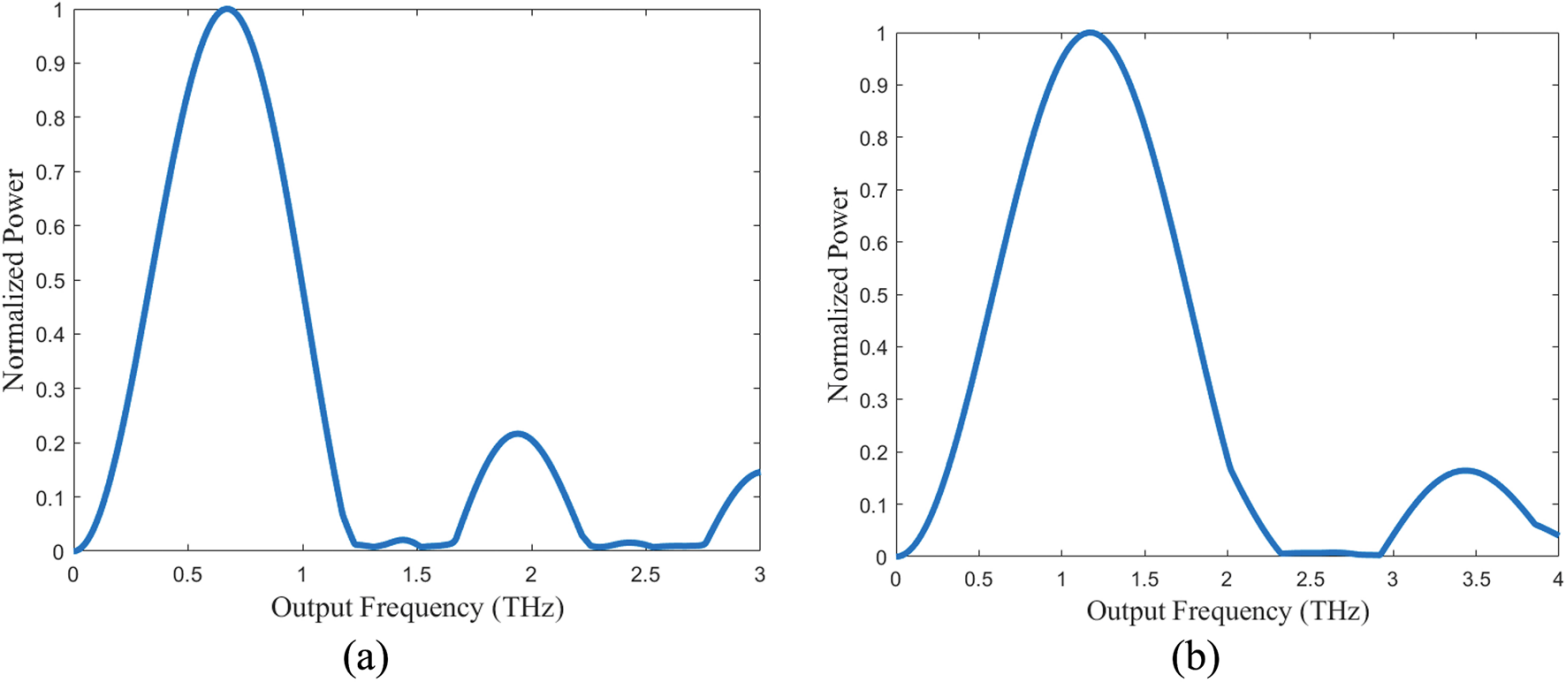
Simulated frequency spectra for the modified rotating dipole model. (**a**) Frequency spectrum of the simulated 0.5 THz system. A peak emission is observed at 0.67 THz. (**b**) Frequency spectrum of the simulated 1 THz system. A peak emission is observed at 1.17 THz.

**Figure 6:**
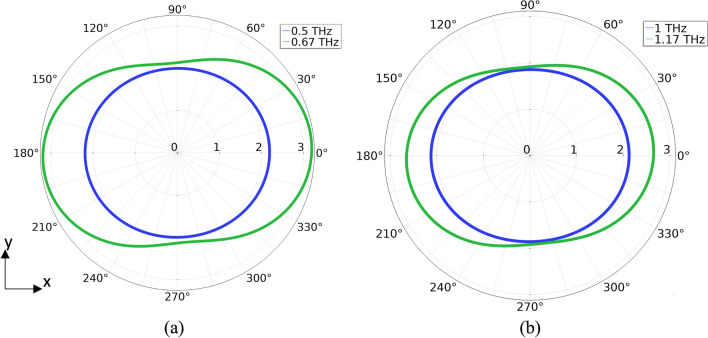
Simulated radiation patterns for the modified rotating dipole model (arbitrary scale) in the orbital plane. (**a**) Radiation pattern for the 0.5 THz system. The design frequency of 0.5 THz (blue) radiates uniformly in all directions in the plane of rotation. The peak frequency 0.67 THz (green) radiates in all directions in the orbital plane, but is more biased along the x-axis. (**b**) Radiation pattern for the 1 THz system. The design frequency of 1 THz (blue) radiates uniformly in all directions in the plane of rotation. The peak frequency of 1.17 THz radiates in all directions of the orbital plane, but is biased along the x-axis.

The simulations for the dipole model agree with the analytical model in that the target frequency is achieved. However, the peak power emission is at a frequency greater than the target. For the 0.5 THz system, the peak power occurs at 0.67 THz (Fig. 5). For the 1 THz system, the peak power occurs at 1.17 THz. Like the analytical studies, additional peaks at about 3 times the target frequency are present. In terms of spatial distribution, the target frequency emits uniformly in the plane of rotation in agreement with the theoretical calculations (Fig. 6). The peak frequency emits in all directions but is more biased along the x-axis. From the steady state dipole simulations, the 0.5 THz system emits a total power of 15.55 pW and the 1 THz system emits a total power of 25.4 pW. The simulated steady state dipole power emissions for both cases slightly overestimate the analytical results of 13.8 pW for the 0.5 THz system and 22.24 pW for the 1 THz system.

### Arc model

**Figure 7:**
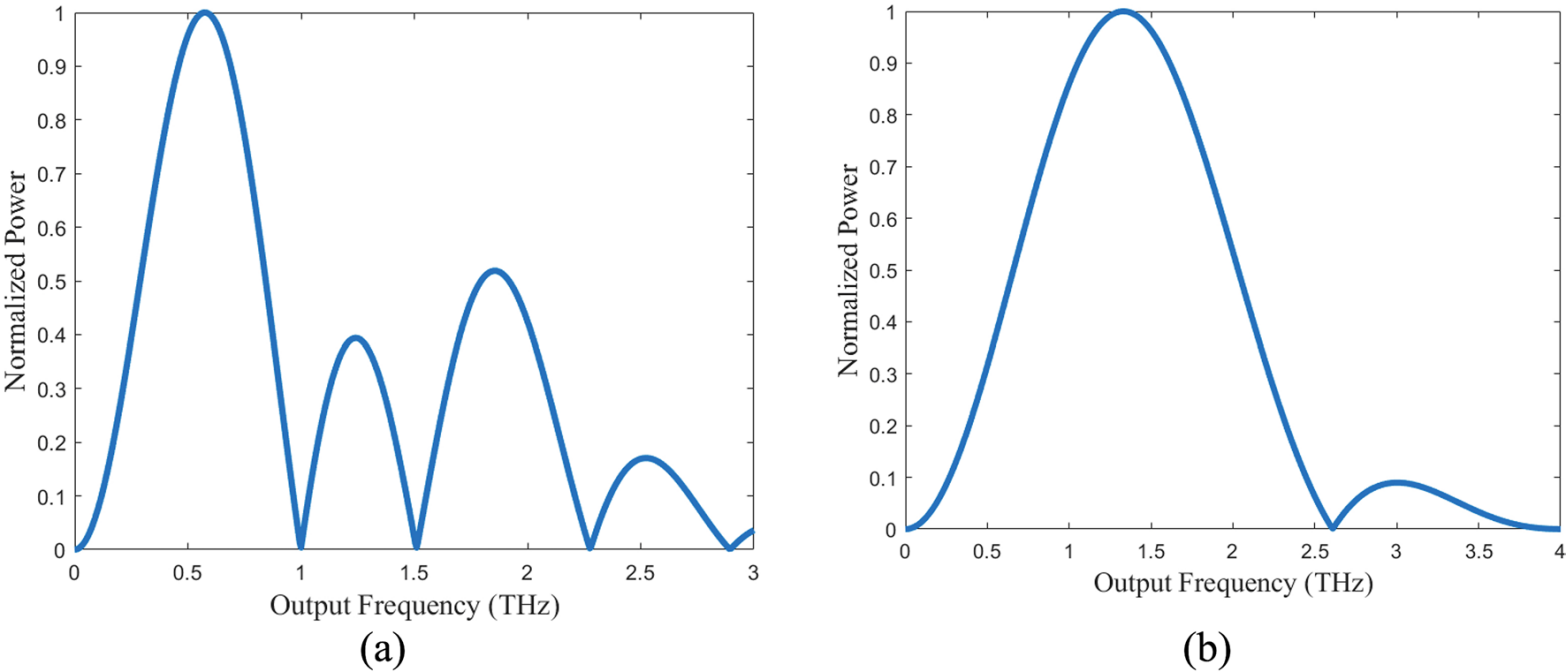
Simulated frequency spectra for the non-equilibrium transport arc model. (**a**) Frequency spectrum of the 0.5 THz system. A peak occurs at 0.57 THz with secondary peak occurring at 1.8 THz. A tertiary peak occurs at 1.25 THz, previously not predicted by the dipole model. (**b**) Frequency spectrum of the 1 THZ system. A peak occurs at 1.33 THz with a secondary peak at 3 THz.

**Figure 8:**
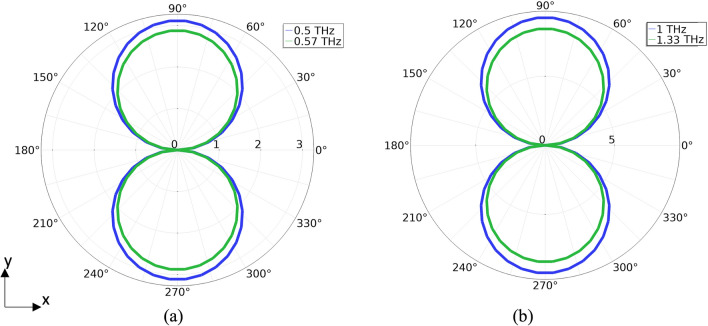
Simulated radiation patterns for the non-equilibrium transport arc models in the orbital plane (arbitrary scale). (**a**) The 0.5 THz system and the 1 THz system (**b**) at the design frequency (blue) and at peak emission (green). The finite element software used does not account for particle dynamics and hence does not produce the expected circular rotating dipole radiation pattern. The shape instead resembles a dipole oscillating along the x-axis.

The arc simulations were performed to determine for the effect of finite device width. For both designs, the target frequencies of 0.5 THz and 1 THz are achieved (Fig. 7). For the 0.5 THz design, the peak power is emitted at 0.57 THz, while the 1 THz design’s peak power is emitted at 1.33 THz. Total power is calculated to be 18.4 pW for the 0.5 THz arc and 13.8 pW for the 1 THz arc. Due to limitations in COMSOL’s capabilities, the radiation patterns for the arcs did not capture the particle dynamics of the charge carriers in the arc (Fig. 8). As a result, the expected radiation pattern from a rotating charge is not produced by the simulations. The shape instead resembles that of a dipole oscillating along the x-axis.

**Figure 9:**
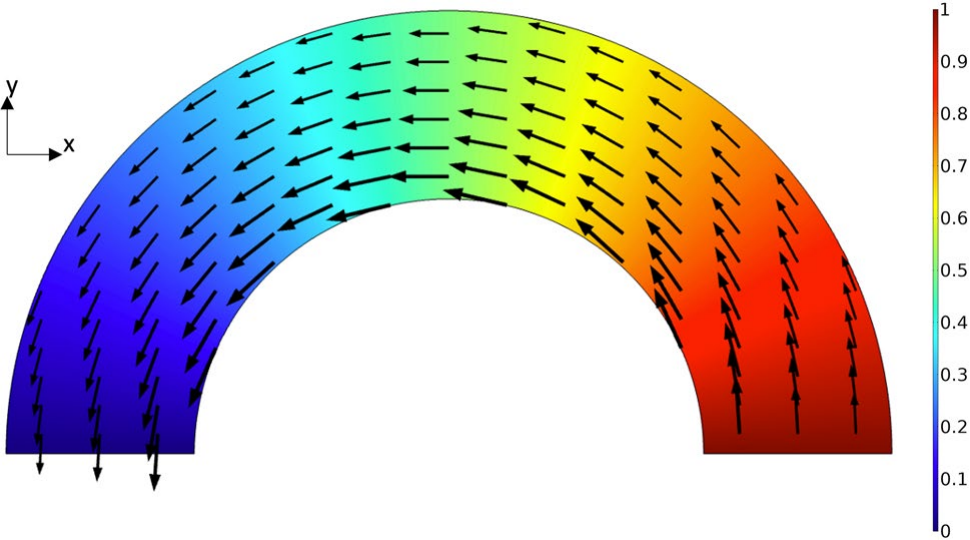
Simulated current density under DC bias of 1 V potential difference between source and drain. In both systems, current flows along the arc with the highest current density at the inner radius as indicated by arrow size. This is unlike the point charge assumption in which all of the charge located at the inner radius.

To illustrate the deviation from the point charge models, DC simulations were performed on the respective arcs (Fig. 9). The simulations with an applied bias of 1 V indicate that current flows from the source, on the right hand side, to the drain, on the left, along the field gradient. The current is most closely concentrated towards the inner radius as indicated by arrow length and thickness. Unlike the point charge assumption, in the arc, charges are distributed along the width of the device.

**Figure 10:**
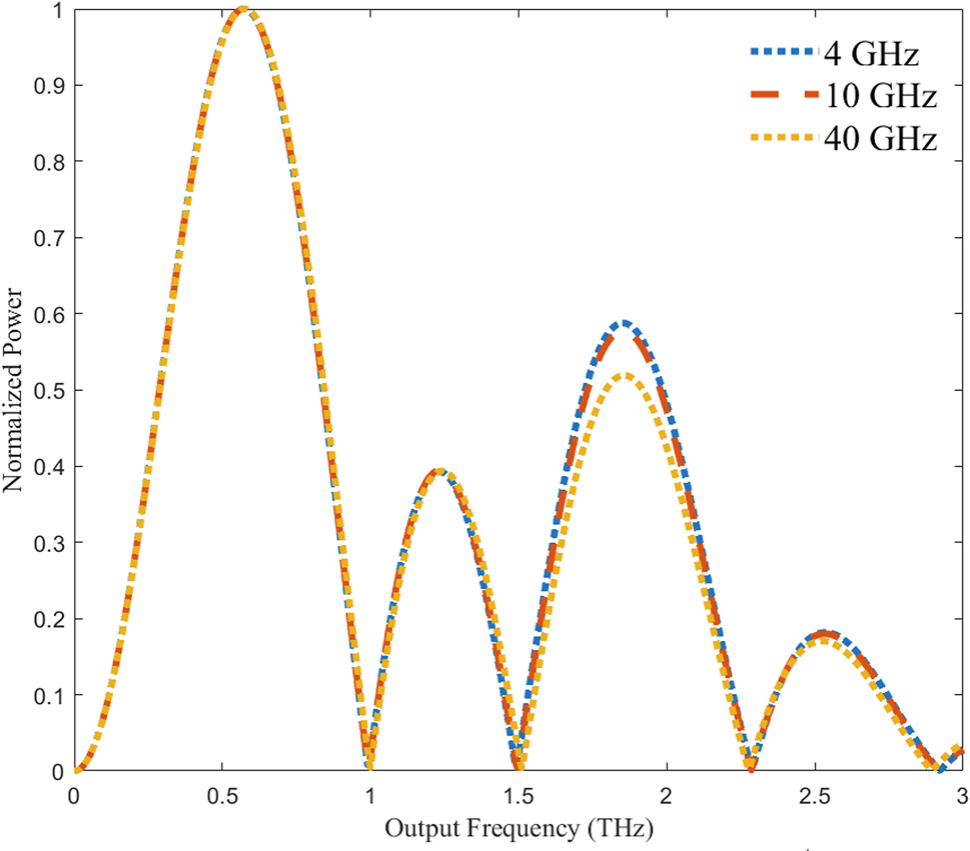
Effect of stimulus frequency on emission spectrum with 4 GHz, 10 GHz, and 40 GHz stimuli. Normalized spectra over the set of applied stimuli for the 0.5 THz design. For all stimuli, the shape is consistent. The 4 GHz and 10 GHz slightly overshoot the 40 GHz secondary peak at 1.8 THz.

Another deviation from the point charge model pertains to device stimulation. For the concept of operation, a pulsed stimulus with a voltage amplitude of 1 V is applied between source and drain terminals. A 40 GHz Gaussian pulse was used in the preceding simulations as it is a sufficiently high frequency that is still attainable with existing technology. With lower frequency stimuli, the frequency spectrum is expected to be degraded with more spurious components being present. The emission spectrum was obtained for additional stimuli at 4 GHz and 10 GHz. Figure 10 shows the spectral response of the 0.5 THz system. Remarkably, the spectrum shape is consistent over all the stimulus frequencies.

The simulation results over a set of different stimulus frequencies [Fig. 10] indicate that the emission spectrum is independent of the stimulus frequency. This result was not expected as it is anticipated that the device would be less responsive at frequencies farther away from the target frequency and thus the emission spectrum would more likely consist of spurious emissions. The device’s emission spectrum is a function of its geometry defined by the arc’s inner radius.

## Discussion

The possibility of terahertz emission from a solid-state cyclotron-radiation emitter device with a graphene substrate has been demonstrated. A simplified an analytical model showed that the device can first be modeled as the classic rotating dipole problem by assuming that all the substrate charges can be treated as a single point charge. Two finite element models were also created to first, verify the analytical results and second, account for the finite dimensions of the graphene arcs. Both output power and spectral characteristics were obtained by all three models. Table 1 shows the calculated output power. For the 0.5 THz system all calculations agree within the same order of magnitude and are very close. For the 1 THz system, the simulated dipole agrees with the Larmor calculation while the simulated arc underestimates by nearly 10 pW. Given the results of both systems, a single graphene solid state cyclotron operating at 0.5–1.0 THz is expected to produce on the order of 10 pW of total radiated power.

**Table 1:** Output power of the graphene nanocyclotrons estimated using 3 different models.

Model	Power (pW)	
	0.5 THz	1 THz
Analytical	13.8	22.0
FE dipole	15.5	25.4
FE arc	18.4	13.8

In terms of radiated frequency spectrum, the transient operating nature of this device implies that even with the point charge assumption that is constrained to orbit a fixed radius, the emission spectrum would be distributed around the angular velocity. This distribution is expected to contain a frequency equivalent to the angular velocity of the orbiting charge with a peak power emission at a frequency ~ 1.36 times the angular velocity. For the 0.5 THz system both the simulated dipole and the simulated arc produce the target frequency of 0.5 THz and their peak emissions occur at 0.67 THz and 0.57 THz respectively. This gives a ratio of 1.34 for the simulated dipole and 1.14 for the simulated arc. For the 0.5 THz system, the simulated dipole approaches the theoretically predicted peak while the simulated arc falls slightly short. The 1 THz system, however, yields a ratio of 1.17 for the simulated dipole and 1.33 for the simulated arc. For both systems, each of their respective simulations slightly underestimate the predicted peak frequency. It was expected that the simulated dipoles would yield the closest values since these approximations are equivalent to the analytical calculations. Given these results, one can expect the peak frequency to be higher than the target frequency between 1.14 and 1.36 times the angular velocity.

With the aforementioned result, one can design a system in which the desired frequency is the peak frequency and not the orbital angular frequency. Such a system could then achieve the same frequency emission with a larger physical footprint than a system in which the angular velocity is the target frequency. This would be advantageous from a prototyping fabrication perspective as the larger footprint would provide additional margin for the processing steps hopefully yielding high fidelity patterns over a design with smaller margins. It is important to mention that, designing for the peak emission will come at the expense of the uniform radiation pattern (Fig. 6).

The predicted device power per area is on the order of 1 nW/cm^2^. This device performance is in the upper range of existing work on graphene wigglers which are expected to emit 1 pW/cm^2^ to 10 nW/cm^2^^13,26^. Such power can be realized with large scale repetition using existing semiconductor processing methods. Given the geometric constraints of a semicircle, it is possible that the desired radiation pattern can be preserved over an array of these devices by having alternating oriented semicircles such that the source and drain terminals also alternate instead of a simple repetition and translation of the semicircle and associated interconnects. Such an implementation would piecewise form a full circle and would save on manufacturing space by reusing a terminal that can be used by two neighboring units.

The results of these simulations are promising enough to fabricate the proposed device. Further simulation work to be performed would focus on increasing device density and associated effects on device performance, namely the effects of the interconnect network on parasitic capacitance.

For any meaningful power to be obtained, large arrays of the device are necessary. To achieve the desired coherence, integration of the device with a photoconductive antenna with external laser stimulus would be an ideal solution due to the superior bandwidth of optical domain over the electrical or RF domain^9^. With recent advancements in telecommunications technology, specifically 5G devices, RF based devices with high enough operating frequencies (10 s of GHz)^27^ applicable in phased array technology^28^ can be integrated with the graphene device to achieve the desired operating conditions. This may be the more desirable solution in terms of cost compared to the laser-based solution due to the soon to be readily commercially available 5G integrated circuits and the associated compact form factor. Such technological developments may allow for a graphene THz emitter system-on-a-chip.

A solid-state implementation of a THz cyclotron radiation source in graphene has been investigated through analytical dipole models and finite element simulation and results indicate that such a device is feasible. Designs in the 0.5–1.5 THz are supported by a semicircular arc geometry and can emit power in the range of 1 nW/cm^2^. With a highly scalable planar semicircular geometry along with existing semiconductor processing methods, this predicted power range can be realized and potentially leading to low-cost THz emitter sources.

## Methods

Finite element simulations of the simple dipole model in both classical continuous rotating formulation and modified semicircuslar conditions followed by a semicircular arc of graphene in steady state RF and non-equilibrium transport conditions were also performed. In COMSOL Multiphysics, a point dipole located in the center of the coordinates was created with charge equivalent to the sum of all free carriers of the graphene arcs that correspond to each target frequency (0.5 THz and 1 THz).

This value is obtained by multiplying the arc area by the charge density. In this study, a charge density of ${10}^{12}$cm^−2^ is used as such a value can be realistically obtained at room temperature while having mean free paths on the order of 1 $\mu$m. For the 0.5 THz system, the effective point charge corresponds to 1164 electrons while the effective point charge for the 1 THz system corresponds to 739 electrons. Using the RF module in COMSOL, the charge starts at a position $\overrightarrow{r}\left(r,\phi ,\theta \right)=({r}_{arc}, 0,\frac{\pi }{2})$ and orbits the origin in a counterclockwise manner. In the steady state condition, the charge is allowed to make complete orbits like in the classic rotating dipole problem. A scattering boundary condition was defined for a 10 $\mu$m radius sphere containing the dipole. This model was used to calculate the maximum power output and is compared to the Larmor power formulations.

In addition, a modified dipole model was developed to capture the frequency spectrum and radiation pattern. Much like the classical model, all the arc’s charge is concentrated to a point and orbits the origin in a counterclockwise fashion at a fixed radius. Unlike the classical model, the modified dipole model is only valid for ½ of an orbital period. The system is expected to emit a range of frequencies in which the target frequency is emitted uniformly in the plane of rotation. According to the analytical results (Fig. 3) a peak emission at a frequency 36% higher than the target frequency is also expected.

More realistic models were developed to help prove the concept, where a finite arc width (100 nm) is considered. The graphene layer was modeled as a boundary with sheet resistivity specified by the manufacturer 430 $\Omega$/sq^17^. First, using COMSOL’s AC/DC module, a direct current (DC) simulation was performed. The straight edges of the arc were set as ports with fixed potential where one was ground and the other 1 V. This simulation allows for verification of the field gradient and current flow along the arc. Next, a steady state RF simulation of the arcs stimulated at their respective target frequencies is performed to calculate the power output. Lastly, a non-equilibrium transport simulation is performed on the arcs for a time of 1/2 of a full orbital period with a Gaussian pulse stimulus centered at 40 GHz, 10 GHz, and 4 GHz. This stimulus frequencies were chosen as they can be readily obtained with existing technology.
